# Introducing a novel beta-ray sensor based on polycarbonate/bismuth oxide nanocomposite

**DOI:** 10.1038/s41598-022-06544-6

**Published:** 2022-02-15

**Authors:** Seyed Musa Safdari, Shahryar Malekie, Sedigheh Kashian, Morteza Akbari

**Affiliations:** 1 Department of Nuclear Engineering, Science and Research Branch, Islamic Azad University, Tehran, Iran; 2 Radiation Application Research School, Nuclear Science and Technology Research Institute, P.O. Box 31485-498, Karaj, Iran

**Keywords:** Nanoscale materials, Computational methods, Experimental nuclear physics

## Abstract

In this research, for the first time, the polycarbonate/bismuth oxide (PC–Bi_2_O_3_) composite was studied as a beta-ray sensor using a pure beta-emitter ^90^Sr source. Firstly, the range and stopping power of the electrons in the composite at various loadings of 0, 10, 20, 30, 40, and 50 wt% were calculated using the ESTAR program. Results of simulation demonstrated that the concentration of the heavy metal oxide particles into the polymer matrix played an important role in evaluating the range and stopping power of the electrons in the composite. Secondly, at the experimental phase, the pure Polycarbonate and 50 wt% PC–Bi_2_O_3_ nanocomposite with dimensions of 4 × 4 × 0.1 cm^3^ were prepared and irradiated by ^90^Sr. Also, current–voltage (I–V) plot exhibited linear response ranging from 100 to 1000 V at the fixed source‐to‐surface distance (SSD). Then the amount of electric current as the sensor response was measured in various dose rates at the fixed voltage of 400 V for the pure Polycarbonate and 50 wt% PC–Bi_2_O_3_ nanocomposite using an electrometer, in which results showed that the sensitivities were found as 20.3, and 33.3 nC mSv^−1^ cm^−3^, respectively. This study showed that this composite could serve as a novel beta-ray sensor.

## Introduction

Detection and dosimetry of ionizing radiation are important issues in the nuclear industry. Recently, polymer-nanocomposites have been used as radiation sensors, detectors, dosimeters, and shielding materials^1–15^. The interaction mechanisms of beta particles with matter are categorized in two sections, electron excitation and ionization, in which electrons interact with the particles traversing the material via the Coulomb electric field^16–18^. Electrons lose their energies by friction attributed to the CSDA or continuous slowing-down approximation^19–21^. The collisions of electrons with the particles include hard collisions or inelastic scattering with orbital electrons produce excitation or ionization of electrons, and secondary electrons, inelastic scattering with nuclei leads to create Bremsstrahlung, and soft collisions or elastic scattering, in which electrons lose a small fraction of their energies^16^. It is worth pointing out here that ionizing radiations, including electrons through the interaction with the nanostructured materials, due to atomic displacement can alter the crystal structure and induce various events such as emission of secondary electrons, excitation, ionization, and bond breakage of the material atomic structures^22^.

Some radioisotopes decay via beta-minus emission, producing the fast electrons^23^. Several pure beta-emitters are ^3^H (18.6 keV), ^14^C (156 keV), ^32^P (1.71 MeV), ^33^P (248 keV), ^35^S (167 keV), ^36^Cl (714 keV), ^45^Ca (252 keV), ^63^Ni (67 keV), ^90^Sr/^90^Y (546 keV/2.27 MeV), ^147^Pm (224 keV), and ^204^Tl (766 keV)^23^.Various types of scintillators are commonly used to detect beta-rays. In addition, low Z materials, including organic polymers, are excellent absorbers of charged particles such as beta-rays, which will provide high sensitivity for charged particle detection^20,24–27^. A disadvantage of some scintillators and gas-flow type proportional counters is limitations due to their hygroscopicity and scalability^27^.

This research aims to design and fabricate a novel beta-ray solid-state material to detect the beta-emitter sources as a real-time sensor. The material used is novel and uses the polycarbonate/bismuth oxide composite (PC–Bi_2_O_3_).

Several experimental findings indicated that a higher degree of polymeric matrix crystallinity hindered nanoparticle dispersion at higher concentrations^28^, Therefore, in this research, Polycarbonate with an amorphous structure was selected as the matrix. Although in the previous research published by this research group, XRD analysis indicated the semi-crystalline nature of the PC–Bi_2_O_3_ nanocomposite^1^.

Chemical or physical interfacial bonding between nano-fillers and polymer matrix plays an important role in the fabrication process^29^. Polycarbonate is essentially an amorphous polymer expected to have more suitable bonds with the Bi_2_O_3_ nanoparticles. Polycarbonate contains end groups including conjugated double bonds^30^, C=O (carbonyl), C–H, Phenyl, and C–O–C bands^31^. Due to similar polar groups containing oxygen in molecules bound to PC and Bi_2_O_3_ combined through either Van der Waals force, H bond, or other forms of the covalent bond^32,33^.

For the PC–Bi_2_O_3_ composite sensor, the amount of sensitivity or minimum detectable dose rate (MDDR) for detecting the beta-rays can be controlled via the weight fractions of the heavy metal oxide particles in the polymer matrix. Generally, various factors affect the sensor response of a polymer-nanocomposite, including polymer crystallinity, the weight percentage of metal oxide nanoparticles, dispersion state of metal oxide nanoparticles into the polymer matrix, nanocomposite thickness, and other factors.

The selection of suitable thickness for a solid sensor, considering the charge particle equilibrium (CPE), is a key factor for detecting ionizing radiation. So, calculation of range and stopping power of electrons at different energies and weight fractions of the inclusions in a polymer composite using the ESTAR program should be carried out. After evaluating the electron range in the media, it is possible to calculate the exact amount of optimal sensor thickness at certain energies.

Strontium-90 (^90^Sr) was chosen as a pure beta-emitter source in this research. The ^90^Sr source is generated in the reactor by the fission reaction of the ^235^U nuclei as^34^:

1 $$ {}_{92}^{235} {\text{U}} + {}_{0}^{1} {\text{n}} \to \left[ {{}_{92}^{236} {\text{U}}} \right] \to {}_{38}^{90} {\text{Sr}} + {}_{54}^{143} {\text{Xe}} + 3{}_{0}^{1} {\text{n}} $$

The decay mechanism of ^90^Sr is as follows^34^:

2 $$ {}_{38}^{90} {\text{Sr}} \to {}_{39}^{90} {\text{Y}} +\upbeta ^{ - } + {\overline{\upnu }} \to {}_{40}^{90} {\text{Zr}}({\text{stable}}) +\upbeta ^{ - } + {\overline{\upnu }} $$

^90^Sr decays to ^90^Y with a half-life of 28.78 years, with beta particle energy of 546.2 keV; the ^90^Y, which emits beta and gamma, converts to ^90^Zr with a half-life of 64 h, with beta particle energy of 2.28 MeV^34^.

In this research, for the first time, a novel beta-ray sensor based on the PC–Bi_2_O_3_ nanocomposite was introduced, and the sensor response of this material was investigated theoretically and experimentally to a pure beta particle source ^90^Sr. The variation of electric current (net current) during the irradiation was considered as the sensor response.

## Materials and methods

### Sample preparation

**Figure 1:**
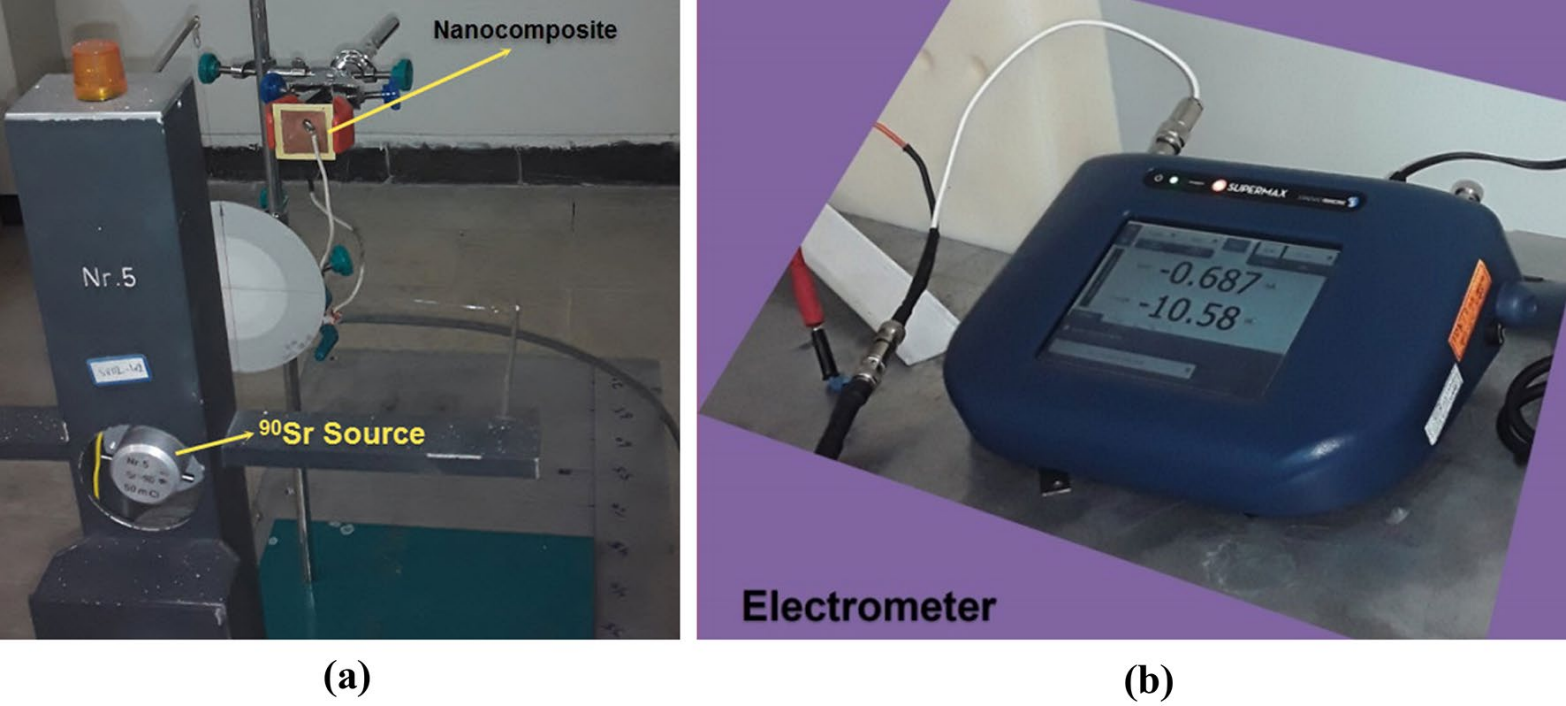
Measurement setup for sensing the beta-rays using (**a**) beta irradiation system (Buchler) and (**b**) Supermax Standard Imaging electrometer.

Pure Polycarbonate and 50 wt% PC–Bi_2_O_3_ nanocomposite were synthesized using the solution casting method. The synthesis details have been described in our previous work^1^. PC as a polymer matrix and Bi_2_O_3_ nanoparticles with the average size of 90–210 nm as nano-fillers were used with densities of 1.2 g cm^−3^ and 8.9 g cm^−3^, respectively. In this experimental work, to irradiate the samples, as exhibited in Fig. 1a, a beta irradiation system model Buchler BSS-BA containing a ^90^Sr reference source with an initial activity of 50 mCi (production date 1978) which is located in Secondary Standard Dosimetry Laboratory (SSDL) Karaj–Iran was used at different source-surface distances (SSDs) according to Table 1. Also, as depicted in Fig. 1b, the Supermax Standard Imaging electrometer was used to measure the electric charge during irradiation at fixed time steps of 15 s. To fabricate the electrodes on two surfaces of the top and bottom of the samples, the copper sheets with areas of 3.5 × 3.5 cm^2^, and 4 × 4 cm^2^, and the thickness of 100 µm were attached to the top and bottom surfaces of the samples respectively using the silver paste.

**Table 1:** The amounts of SSDs for ^90^Sr and corresponding dose rates.

SSD (cm)	Dose rate (mSv h^−1^)
30	102.436
35	75.259
40	57.620
45	45.527
50	36.877
55	30.477

Prior to the beta irradiation process, some control experiments, including electrical characterization, should be performed on the pure polymer and the nanocomposite samples. The electrical conductivity of the sensor material plays an important role in sensor performance. By applying a suitable bias voltage to the samples, the amount of dark current should be at the level of pico-ampere; therefore, the insulation test of the samples should be performed before the beta irradiation in order to prevent the electroforming phenomenon (migration of metal particles to the sensitive volume of the sensor)^14^. Thus, in this research, in order to ensure the insulation of the samples, a Digital Insulator Tester MIS-3D was used.

It should be mentioned that electric charges are measured using the electrometer at the specified voltages during fixed time steps of 15 s. Therefore, the amount of electric current passing through the radiation sensor is obtained by dividing the measured electrical charge by the collection time.

**Figure 2:**
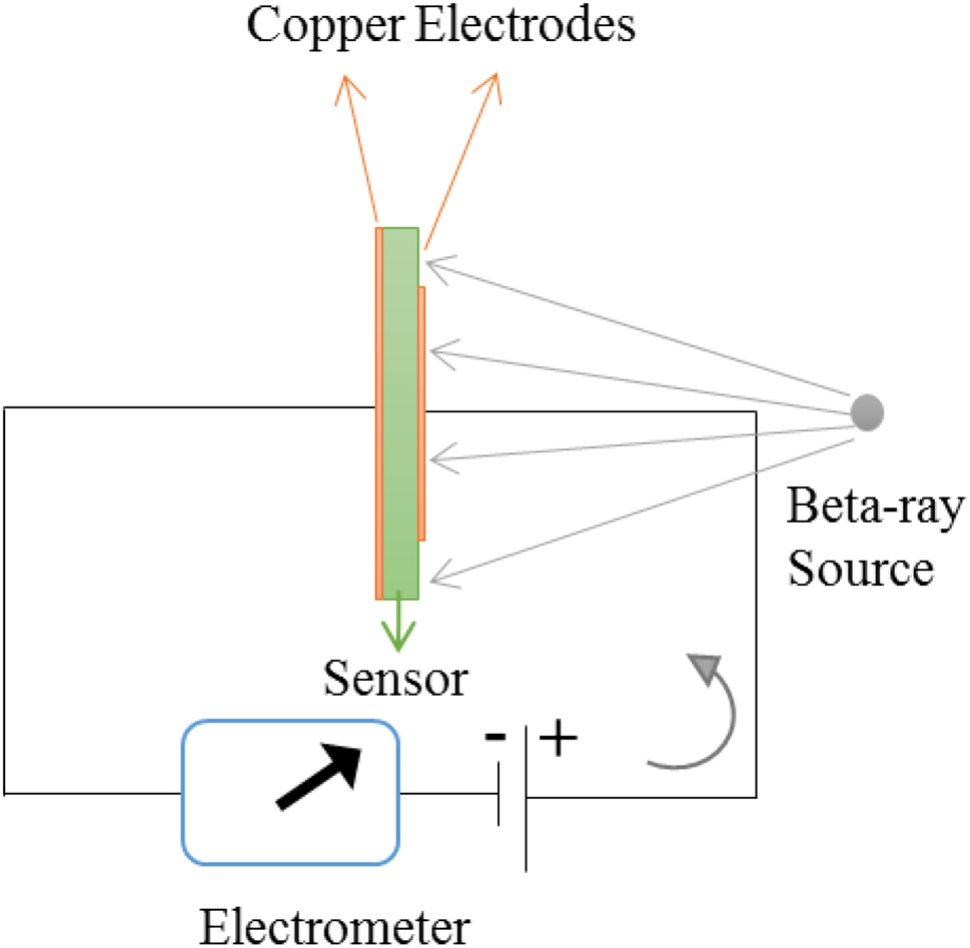
Electric circuit designed for sensing the beta-rays using the PC–Bi_2_O_3_ nanocomposite.

In Fig. 2, the electric circuit for sensing the beta-rays is depicted using the PC–Bi_2_O_3_ nanocomposite sensor. During the fabrication of the electrodes, as can be easily seen from Figs. 1a and 2, the area of the copper electrode on the surface in front of the incident beta-rays was considered as 3.5 × 3.5 cm^2^. In contrast, the surface of the sample was 4 × 4 cm^2^; thus, the top copper electrode did not cover the entire material surface, and a part of the sample surface was directly exposed to the beta-rays.

### Simulation methodology

In this research, a pure beta-emitter of ^90^Sr with two energies at 546.2 keV and 2.28 MeV was chosen to investigate the sensor response of the PC–Bi_2_O_3_ nanocomposites to beta-rays. To have an insight into the range and stopping power of the produced electrons in the composite via the beta irradiation at different weight fractions of the heavy metal oxide fillers, we have calculated these quantities using the ESTAR program^35^. The performance of any ionizing radiation detector sensor essentially depends on the approach in which the radiation to be detected interacts with the material of the detector sensor itself^23^. The linear stopping power for charged particles in a particular absorber is defined as the energy loss for that particle within the material divided by the corresponding path length via the Bethe–Bloch formula^23^:

3 $$ - \frac{{{\text{dE}}}}{{{\text{dx}}}} = \frac{{4\uppi {\text{e}}^{4} {\text{z}}^{2} }}{{{\text{m}}_{0} {\text{v}}^{2} }}{\text{NZ}}\left[ {\ln \frac{{2{\text{m}}_{0} {\text{v}}^{2} }}{{\text{I}}} - \ln \left( {1 - \frac{{{\text{v}}^{2} }}{{{\text{c}}^{2} }}} \right) - \frac{{{\text{v}}^{2} }}{{{\text{c}}^{2} }}} \right] $$

In which v, ze are the velocity and charge of the particles, N and Z are the number density and the atomic number of the absorber atoms, m_0_ is the electron rest mass, e is the electronic charge, and I indicates to average ionization potential of the absorber material^23^.

## Results and discussion

### FESEM analysis

**Figure 3:**
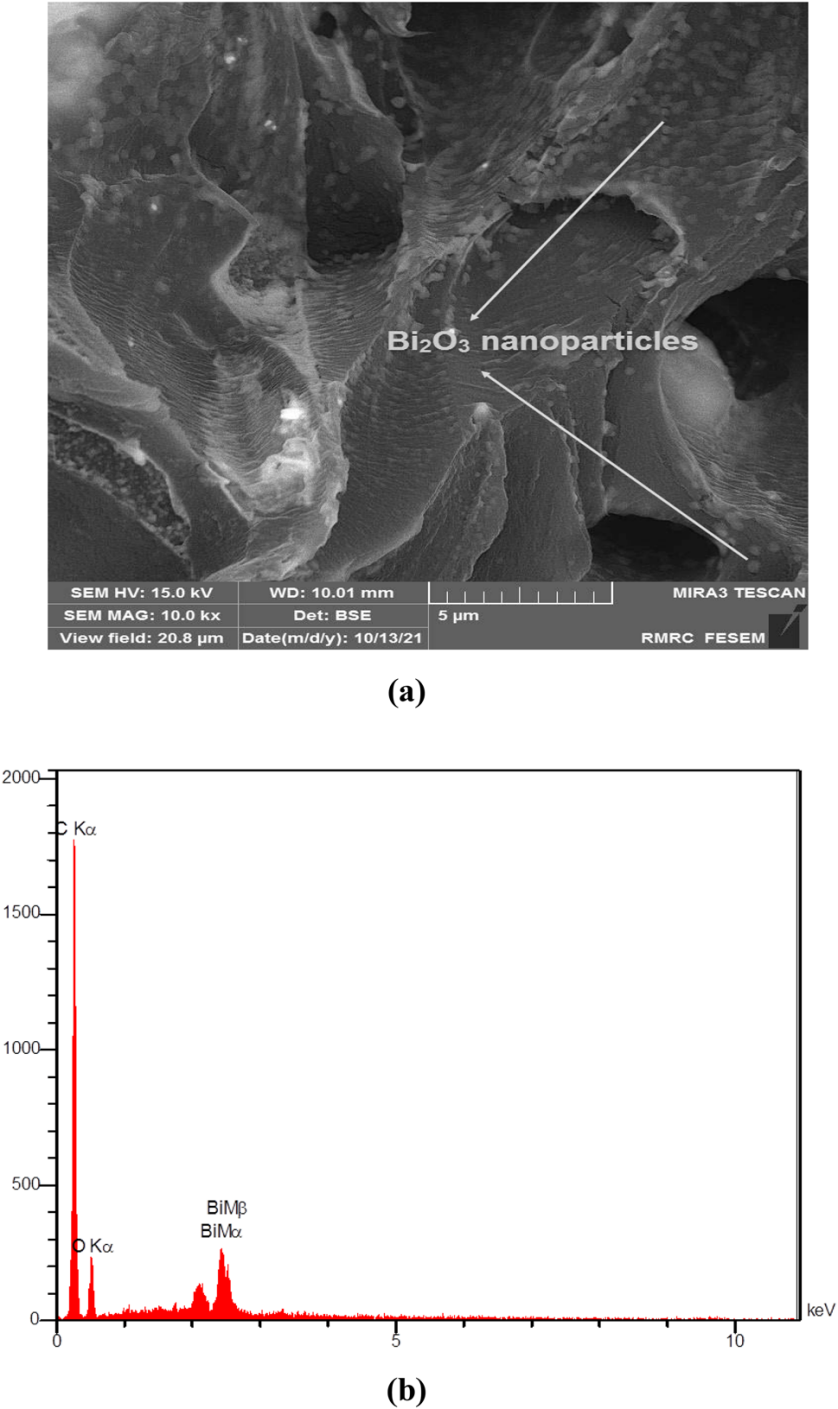
Illustration of (**a**) FESEM of the fractured surface, and (**b**) EDS analysis corresponding to the 50 wt% PC–Bi_2_O_3_ nanocomposite.

The surface morphology of the prepared 50 wt% PC–Bi_2_O_3_ nanocomposite was analyzed by Field Emission Scanning Electron Microscopy (FESEM) and Energy-Dispersive X-ray Spectroscopy (EDS). These were conducted at the Razi Metallurgy Research Centre in Iran. For this purpose, the FESEM device model MIRA3TESCAN-XMU was used. As can be observed in Fig. 3a, a cross-sectional view of the sample showed an appropriate dispersion state of the Bi_2_O_3_ nanoparticles into the PC matrix. Maybe at higher concentrations of the Bi_2_O_3_ nanoparticles, agglomeration and aggregation have emerged due to inter-particle interactions, affecting this nanocomposite radiation sensing performance^13,36^.

Figure 3b exhibits EDS analysis of the 50 wt% PC–Bi_2_O_3_ nanocomposite to identify the constituent elements of this material. As shown in Fig. 3b, EDS analysis confirmed the presence of Bi, O, and C elements in this nanocomposite.

### Results of simulation

**Figure 4:**
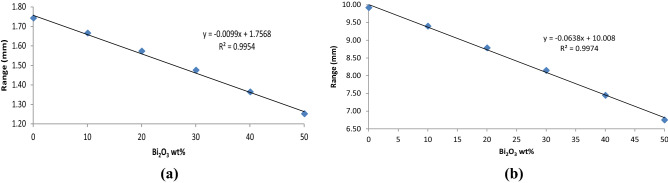
Calculation of electrons range versus Bi_2_O_3_ wt% for electrons at energies of (**a**) 546.2 keV and (**b**) 2.28 MeV using the ESTAR program in the PC–Bi_2_O_3_ composite.

As shown in Fig. 4, simulation results of the beta-emitter source of ^90^Sr at two energies of 546.2 keV and 2.28 MeV for the 50 wt% PC–Bi_2_O_3_ composite at various weight fractions are depicted. It can be mentioned that increasing the weight fraction of the Bi_2_O_3_ particles into the Polycarbonate matrix lead to a linear decrease of the range of beta particles in the composite material. This phenomenon is probably due to the increasing the probability of Bremsstrahlung secondary radiation by adding the Bi_2_O_3_ wt% into the polymer matrix. The energy dissipation of incident beta particles via interaction with this composite material is increased by Bremsstrahlung radiation. Thus the range of the particles will be decreased subsequently.

The simulation results showed that the optimal thickness for detecting predominant beta particles of 546.2 keV in ^90^Sr source for the 50 wt% PC–Bi_2_O_3_ composite was estimated to be approximately 1.2 mm. It seems that a thickness of 1 mm for this sensor would be suitable for measuring beta-rays with the energy of 546 keV, and also it can be mentioned that the CPE phenomenon would be established. However, detecting the higher energy electrons, including 2.28 MeV, will contribute to relatively poor efficiency. In order to improve the efficiency of a sensor, it is possible to increase the thickness of the material; nevertheless, at higher thicknesses, the applied electric field will be decreased, and it will practically lose its uniformity which leads to reduced sensitivity of the sensor. Thus, selecting the optimal thickness for this sensor is essential.

It is worth pointing out here that the two parameters of size and dispersion state of the Bi_2_O_3_ nanoparticles into the polymer matrix can affect the performance of this sensor consequently. In the previous study by this research group, it was shown that adding the heavy metal oxide nanoparticles with a higher surface-to-volume ratio into a polymer matrix in comparison with micro ones leads to increasing the photon absorption cross-section of the composite subsequently^37^. Considering this effect, the production of X-rays related to the Bremsstrahlung radiation in this sensor can be increased. So, it is expected that the sensor response (electric current) can be further improved if the size of the Bi_2_O_3_ nanoparticles are reduced. Intaniwet et al.^13^, stated that since the Bi_2_O_3_ nanoparticles are electrical insulating, so agglomeration leads to partial blockage of the transport of charge carriers in the polymer-nanocomposite. So the effects of size and dispersion state of the Bi_2_O_3_ nanoparticles into the polymer matrix can be investigated to gain more insights into this issue, and there is a necessity for future research on these topics.

**Figure 5:**
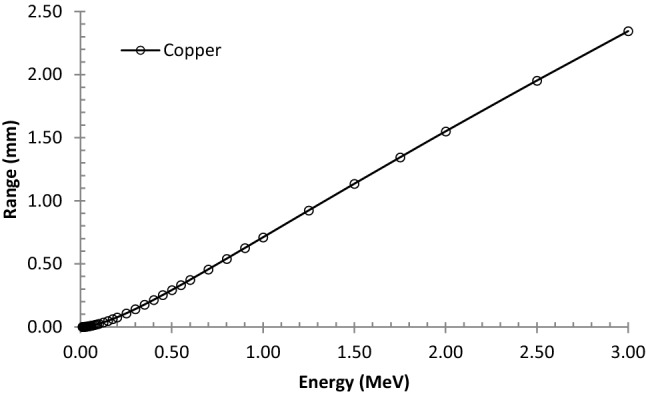
Calculation of the range for electrons at various energies up to 3 MeV in the copper using the ESTAR program.

Since copper electrodes were used in this research, so as shown in Fig. 5, to estimate the effect of electrons range in the pure copper at various energies up to 3 MeV, the ESTAR program was used accordingly. Results showed that the amounts of the range for electrons in the pure copper at two main energies of 546.2 keV and 2.28 MeV were obtained as 330 µm and 1.78 mm, respectively. It seems that energy loss of electrons with energy of 2.28 MeV in the copper electrode with a thickness of 100 µm can be negligible, but for 546.2 keV, this thickness may affect the sensor response. Also, Kasani et al. investigated the influences of ^90^Sr beta-ray irradiation on the electrical characteristics of carbon nanoparticles^38^. Their results through the Monte Carlo simulation using MCNPX code showed that large ratios of beta-ray energies were deposited within the copper electrodes^38^.

In order to obtain better performance for sensing the beta rays, it is recommended that the thickness of the copper electrode in front of the beam should be selected as thin as possible or considered as a fine mesh.

**Figure 6:**
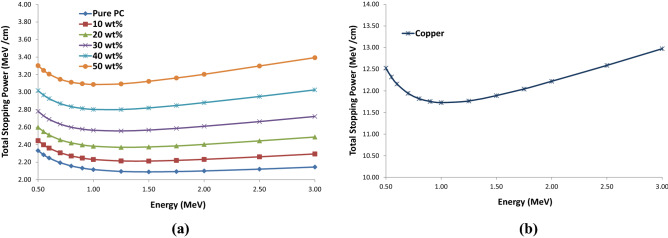
The total stopping power of electrons at different energies up to 3 MeV for (**a**) various weight percentages of the PC–Bi_2_O_3_ composite and (**b**) copper, using the ESTAR program.

Also, according to Fig. 6a, the amounts of total stopping power of the electrons at various energies up to 3 MeV for the PC–Bi_2_O_3_ composite at different weight fractions and the copper electrode were calculated using the ESTAR program. It can be deduced that at the specific and constant energy, increasing the Bi_2_O_3_ wt% will increase the amount of total stopping power of the electrons in the composite. This phenomenon could be attributed to the increment of the effective atomic number of the composite in higher amounts of the reinforcement phase regarding the Bethe–Bloch formula.

Also, according to Fig. 6b, it can be mentioned that the amount of total stopping power of the electrons at various energies up to 3 MeV for the pure copper is significantly more than the PC–Bi_2_O_3_ composite at different weight fractions of the inclusions. Thus, in this investigation, copper electrodes can significantly affect the sensor response.

Previous simulation results showed that increasing the weight fraction of the Bi_2_O_3_ particles into the Polycarbonate matrix led to a linear decrease of the range of beta particles in the composite material and increased the total stopping power regarding the more dissipation charged particles, including Bremsstrahlung radiation. Thus, it could be concluded that the sample with 50 wt% Bi_2_O_3_ exhibited minimum range at two main energies of ^90^Sr beta-ray source namely 546.2 keV and 2.28 MeV. So, it is predictable that the maximum efficiency for sensing response should be attributed to the 50 wt% Bi_2_O_3_ sample.

**Figure 7:**
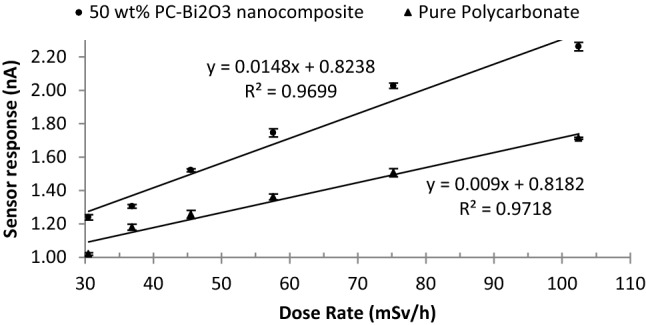
Sensor response of two materials against the ^90^Sr exhibiting a maximum 1.9% standard deviation (1σ).

In Fig. 7, the values of sensor response (net current or I_Net_) under the same conditions using the copper electrodes with the same size at the fixed voltage of 400 V were measured for the Pure Polycarbonate and 50 wt% PC–Bi_2_O_3_ nanocomposite. As can be seen from Fig. 7, adding the Bi_2_O_3_ nanoparticles into the Polycarbonate matrix increases the sensor response significantly at various dose rates. It can be mentioned that the sensor response for the 50 wt% sample was increased gradually from 30 to 75 mSv h^−1^ and eventually showed a tendency to saturate afterward at 102 mSv h^−1^. It seems this saturation is related to the recombination of beta-induced-charged particles, which indicates that the sensor response should be measured at higher voltages. The other reason for the justification of this saturation is related to the issue that the Bremsstrahlung radiation may be affected under a high flux of the electrons (or higher dose rates). Kasani et al.^38^, observed a similar effect related to saturation of the current during the irradiation of carbon nanoparticles by ^90^Sr beta-rays, which may be due to the change of the carrier properties by increasing the scattering of electrons induced at higher dose rates. Also, the scattered electrons via the internal polarization can reduce the electric field in the sensitive volume^5^. In Fig. 7, the correlation coefficients for linear fits are exhibited as R^2^ = 0.999 and R^2^ = 0.9933 for the pure Polycarbonate and 50 wt% PC–Bi_2_O_3_ nanocomposite, respectively.

The electrical conduction in this sensor under beta-irradiation is pertinent to several phenomena. Firstly, the collisions of beta-rays with the orbital electrons in the PC–Bi_2_O_3_ nanocomposite, and copper electrode produce excitation, ionization, and delta-rays (secondary electrons) in the atomic structure of these materials. The probability of this interaction is proportional to the atomic number Z of the absorber material^16^. These secondary electrons can be gathered by applying a suitable voltage to the sensor. Secondly, inelastic scattering with nuclei leads to produce Bremsstrahlung, in which the probability of this interaction is proportional to Z^2^. Interaction of beta-rays with the Bi_2_O_3_ nanoparticles in the nanocomposite and copper electrode can create Bremsstrahlung radiation, especially at the higher flux of the electrons. Finally, elastic scattering between electrons may lose a small fraction of their energies in the material with the probability of Z^2^.

Regarding the mechanism of beta interaction with the PC–Bi_2_O_3_ nanocomposite material, the Bremsstrahlung radiation can be considered a dominant effect due to the presence of the Bi_2_O_3_ nanoparticles. The induced X-ray due to Bremsstrahlung radiation depends on the concentration of the doped Bi_2_O_3_ nanoparticles included in the polymer matrix^13^.

**Figure 8:**
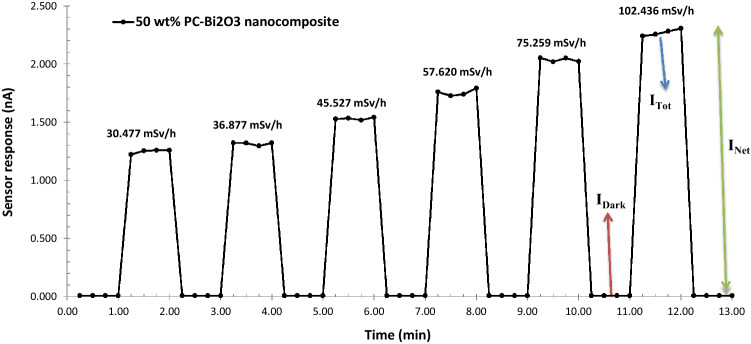
Time evolution of the sensor response at different dose rates for the 50 wt% PC–Bi_2_O_3_ nanocomposite.

In Fig. 8, the time evolution of sensor response, namely electric current at different dose rates for the 50 wt% PC–Bi_2_O_3_ nanocomposite is depicted. As can be seen from this figure, the average initial amount of dark current (current in the absence of exposure at the fixed voltage of 400 V or leakage current) was measured as 0.008 nA or 8 pA. For each measurement, in order to measure the dark current, the electrometer set to zero. Otherwise, maybe the accumulation of the charged particles in the nanocomposite sensor will occur, resulting in a continuous increase of the dark current. Afterward, at SSD = 55 cm with a dose rate of 30.477 mSv h^−1^, the average current value at the fixed voltage of 400 V was measured as 1.248 nA. However, as can be seen from Fig. 8, the amount of current measured at different dose rates increases by 146 to 267 times compared to the dark current. It means that the sensor response (Net current) is measured by subtracting the dark current (in the absence of radiation) from the total current, namely I_Net_ = I_Tot_ − I_Dark_.

Here, the mechanism responsible for the increase in the sensor response (net current) through the beta-irradiation is explained. Beta-irradiation of a polymer-metal oxide composite leads to the transfer of ionizing radiation energy to the composite material, followed by the production of secondary electrons^39^. These secondary electrons can be gathered directly by applying a suitable voltage to the copper electrodes. In the other scenario, it can be mentioned that interactions of secondary electrons with heavy metal nuclei of the bismuth and also copper electrode may result in the creation of Bremsstrahlung radiation, in which a part of induced X-rays can be absorbed in the composite subsequently. In addition, higher concentrations of Bi_2_O_3_ in the polymer matrix exhibit a contribution of Bremsstrahlung radiation. These mechanisms increase the electric current through the sample during beta-irradiation.

**Figure 9:**
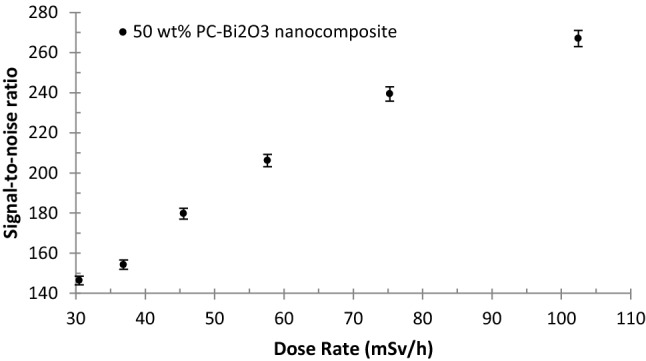
Signal-to-noise ratio of the 50 wt% PC–Bi_2_O_3_ nanocomposite at 400 V against the beta-rays of ^90^Sr with maximum standard deviation of 3.8% (1σ).

One of the most important quantities in determining the sensitivity of a sensor is the signal-to-noise ratio. This quantity is obtained by dividing the net current by the dark current, namely I_Net_/I_Dark_. It is worth mentioning that in this research, the dark current and net current values were measured at the order of pA, and nA respectively at a fixed voltage of 400 V. In Fig. 9, the signal-to-noise ratio of the 50 wt% PC–Bi_2_O_3_ nanocomposite is exhibited. As can be seen from this figure, with increasing the dose rate ranging from 30 to 102 mSv h^−1^, the signal-to-noise ratio will be increased by 146 to 267 times compared to the dark current. This phenomenon may be pertinent to the emission of secondary electrons, excitation, ionization, Bremsstrahlung radiation, and bond breakage during the interaction of the beta particles with the atomic structures of this nanocomposite^37^.

The sensitivities of two materials namely the pure PC and the 50 wt% PC–Bi_2_O_3_ nanocomposite to pure beta-emitter ^90^Sr source were obtained at a fixed voltage of 400 V by dividing the slope of the I-Dose rate plots in Fig. 7 by the active volume of the sensor material (4 cm × 4 cm × 0.1 cm)^13^. The amount of sensitivity of the pure PC and the 50 wt% PC–Bi_2_O_3_ nanocomposite were evaluated as 20.3, and 33.3 nC mSv^−1^ cm^−3^ respectively. Upon beta-irradiation with ^90^Sr, the sensor based on the 50 wt% PC–Bi_2_O_3_ nanocomposite exhibited a sensitivity increase of approximately 1.6 times compared to the pure Polycarbonate sensor.

Here, we compare the results of sensitivity in this research work with other sensors or detectors carried out by other groups. Mills et al.^40^, obtained the sensitivity of a semiconducting polymer diode for the 6 MV X-rays from a medical linear accelerator from 13 to 20 nC mGy^−1^ cm^−3^, for operating voltages from − 50 to − 150 V, respectively^40^. Also, Intaniwet et al.^13^, measured the sensitivity of a semiconducting polymer for 17.5 keV X-ray beam from 10 to 200 nC mGy^−1^ cm^−3^ at 10–200 V, respectively^13^. These results revealed that addition of high-Z Bi_2_O_3_ nanoparticles to a polymer matrix improves the sensor response significantly by enhancing the stopping power of the active volume of the nanocomposite, and creating Bremsstrahlung radiation, especially at a higher flux of the electrons.

**Figure 10:**
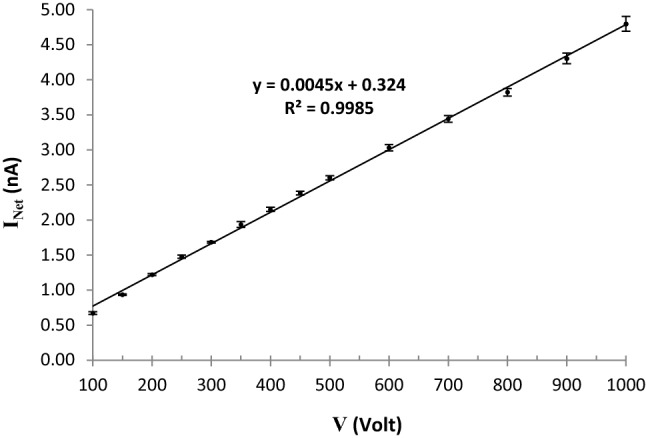
I–V plot of the 50 wt% PC–Bi_2_O_3_ nanocomposite against a ^90^Sr at the fixed SSD = 30 cm and dose rate of 102.436 mSv h^−1^, with maximum standard deviation of 3.8% (1σ).

Generally, finding the suitable operating or working voltage plays an important role in performance of a radiation sensor. In Fig. 10, the current–voltage (I–V) plot of the sensor based on the 50 wt% PC–Bi_2_O_3_ nanocomposite against the beta radiation field of a ^90^Sr source at the fixed SSD = 30 cm and dose rate of 102.436 mSv h^−1^ is exhibited, in which I_Net_ is net current in terms of nA, which is measured by the electrometer. It is obvious from Fig. 10 that the sensor response (net current) is linear at various voltages ranging from 100 to 1000 V. This linearity means that there is no saturation in the sensor response of this nanocomposite up to 1000 V. So it is possible to choose a suitable operating voltage to achieve a significant sensitivity to detect the beta-rays efficiently.

We derived uncertainties type-A from the standard deviation (1σ) of series of measurements via the statistical method:

4 $$\upsigma = \sqrt {\frac{{\sum {\left( {{\text{I}}_{{\text{i}}} - {\overline{\text{I}}}} \right)^{2} } }}{{\text{n}}}} $$

In which $I_{i}$, ${\overline{\text{I}}}$ and n are electric current of i_th_ measurement, the average value of electric current, and the number of measurements respectively. It should be noted that each measurement was repeated four times in time steps of 15 s.

## Conclusion

In this research, for the first time, the sensing response of a novel beta-ray sensor based on the polycarbonate/bismuth oxide composite was studied via the simulation and experiment. At the simulation phase, the range and stopping power of electrons related to ^90^Sr beta-emitter were calculated for various Bi_2_O_3_ wt% in the PC–Bi_2_O_3_ composites up to 50 wt% using the ESTAR program. Results of simulation showed that the amount of heavy metal oxide inclusions in the polymer matrix had a substantial influence on the range and stopping power quantities of the electrons in the material. So, increasing the weight fraction of the Bi_2_O_3_ particles in the Polycarbonate matrix led to a decrease in the range of beta particles and increased the amount of total stopping power of the composite sensor linearly. So, the optimal thickness to detect the main beta particles with energy of 546.2 keV in the ^90^Sr source for 50 wt% PC–Bi_2_O_3_ composite was estimated to be approximately 1.2 mm.

The nanocomposites at loadings of 0 and 50 wt% were prepared at the experimental phase. Then, these samples were irradiated by a beta-emitter of ^90^Sr source. Then, the amounts of electric current passing through the samples were measured using an electrometer at various voltages ranging from 100 to 1000 V exhibiting a linear response. Also, the sensor exhibited a linear behavior at various dose rates ranging from 30 to 102 mSv h^−1^ for the pure Polycarbonate and 50 wt% PC–Bi_2_O_3_ nanocomposite at the fixed voltage of 400 V.

Upon beta-irradiation with ^90^Sr, the sensor based on the 50 wt% PC–Bi_2_O_3_ nanocomposite exhibited a sensitivity increase of approximately 1.6 times compared to the pure Polycarbonate sensor. These results revealed that the addition of high-Z Bi_2_O_3_ nanoparticles to a polymer matrix improves the sensor response significantly by enhancing the total stopping power of the active volume of the nanocomposite, and creating Bremsstrahlung radiation, especially at a higher flux of the electrons.

This exploration showed that the cost–benefit polycarbonate/bismuth oxide nanocomposite could be considered a novel real-time beta-ray sensor to be used in radioactive monitoring systems for medical and industrial applications.
